# Prayer and Medicine

**Published:** 1877-01

**Authors:** Claude Florence


### PRAYER AND MEDICINE.

A minister stood in his pulpit
And preached to the people one day,
"No matter what trouble was on them,
If they would only patiently pray,
That everything wrong would be righted."
This was his doctrine; and then
The choir awoke from their napping
And grandly responded "Amen."

But once we were standing and watching
The minister's wife, sick in bed;
The minister weeping and praying,
The doctor was bathing her head.
There was wailing and moaning and groaning,
Her life it seemed creeping away;
Yet the minister true to his mission,
Had knelt and continued to pray.

And while they kept on with their praying,
The doctor kept mixing away,
And pouring down "fixings" and "physics"—
For doctors ar'n't much on "the pray"—
Well! somehow there wasn't a funeral,
And maybe 'twas owing to prayer;
But I think they'd have purchased a coffin,
Had not that physician been there.

—*Claude Florance.*

